# Estrogen inhibits autophagy and promotes growth of endometrial cancer by promoting glutamine metabolism

**DOI:** 10.1186/s12964-019-0412-9

**Published:** 2019-08-20

**Authors:** Wen-Jie Zhou, Jie Zhang, Hui-Li Yang, Ke Wu, Feng Xie, Jiang-Nan Wu, Yan Wang, Li Yao, Yan Zhuang, Jiang-Dong Xiang, Ai-Jun Zhang, Yin-Yan He, Ming-Qing Li

**Affiliations:** 1 Center of Reproductive Medicine of Ruijin Hospital, Shanghai Jiao Tong University School of Medicine, No.197, Ruijin 2nd Road, Shanghai, 200025 People’s Republic of China; 2 NHC Key Lab of Reproduction Regulation (Shanghai Institute of Planned Parenthood Research), Hospital of Obstetrics and Gynecology, Fudan University, No.1326, Pingliang Road, Shanghai, 200080 People’s Republic of China; 3 Department of Obstetrics and Gynecology, Shanghai General Hospital, Shanghai Jiao Tong University School of Medicine, No.100, Haining Road, Shanghai, 200080 People’s Republic of China; 4 Insititue of Obstetrics and Gynecology, Hospital of Obstetrics and Gynecology, Fudan University, Shanghai, 200032 People’s Republic of China; 5 Clinical Epidemiology, Hospital of Obstetrics and Gynecology, Fudan University, Shanghai, 200011 People’s Republic of China; 6 Shanghai Key Laboratory of Female Reproductive Endocrine Related Diseases, Hospital of Obstetrics and Gynecology, Fudan University, Shanghai, 200011 People’s Republic of China

**Keywords:** Uterine endometrial cancer, CB-839, Estrogen, Autophagy, Glutamine

## Abstract

**Background:**

Excessive estrogen exposure is an important pathogenic factor in uterine endometrial cancer (UEC). Recent studies have reported the metabolic properties can influence the progression of UEC. However, the underlying mechanisms have not been fully elucidated.

**Methods:**

Glutaminase (GLS), MYC and autophagy levels were detected. The biological functions of estrogen-MYC-GLS in UEC cells (UECC) were investigated both in vivo and in vitro.

**Results:**

Our study showed that estrogen remarkably increased GLS level through up-regulating c-Myc, and enhanced glutamine (Gln) metabolism in estrogen-sensitive UEC cell (UECC), whereas fulvestrant (an ER inhibitor antagonist) could reverse these effects. Estrogen remarkably promoted cell viability and inhibited autophagy of estrogen sensitive UECC. However, CB-839, a potent selective oral bioavailable inhibitor of both splice variants of GLS, negatively regulated Gln metabolism, and inhibited the effects of Gln and estrogen on UECC’s growth and autophagy in vitro and / or in vivo.

**Conclusions:**

CB-839 triggers autophagy and restricts growth of UEC by suppressing ER/Gln metabolism, which provides new insights into the potential value of CB-839 in clinical treatment of estrogen-related UEC.

## Background

Uterine endometrial carcinoma (UEC) is a leading cause of female morbidity and mortality worldwide [1]. Surgery at present is satisfactory for treating early-stage UEC; however, the daunting task is to find efficient therapies for advanced UEC patients, who usually suffer from poor prognosis [2], especially in terms of metastatic or recurrent UEC [3]. The 5-year survival rate of localized-UEC patients exceeds 90% after hormonal treatment and hysterectomy, during which process combined chemotherapy and radiation are also proved efficient [4]. Meanwhile, the 5-year survival rate of patients diagnosed with distant metastasis is below depressing 20% because of the lack of efficient available therapies [5]. Therefore, it is important to explore the cellular and molecular mechanisms related to the progression and tumorigenesis of UEC, identify novel therapeutic strategies and search for new diagnostic and prognostic markers for UEC.

The results of previous clinical, biological and epidemiological studies have all demonstrated that excessive and/or prolonged exposure to unopposed estrogen increased the risk of UEC, especially that of the endometrioid type [6]. Two specific intracellular receptors, estrogen receptor (ER) α and ERβ that mediate the biological effects of estrogen actions, regulate cell growth and distinguish a variety of normal tissues from hormone-responsive tumors through interactions with cellular factors [7]. However, the mechanisms of these factors contributing to the malignant state remain unclear despite a growing understanding of the pathophysiology and molecular biology of ERs [8]. Estrogen has also been reported to regulate enzymatic activity in many kinds of cancers. Pastori et al. stated that estrogen induced ribonuclease activity in liver [9]. Moreover, Obayashi et al. elaborated that estrogen controlled branched-chain amino acid catabolism in female rats [10]. However, it is unclear if estrogen regulates the metabolism of UEC by targeting enzyme activity, especially amino acid metabolism.

L-Glutamine (Gln) is an abundant amino acid and plays a vital role in both humans and animals [11]. Physiologic concentration of Gln has been reported to increase autophagy by inactivating mammalian target of rapamycin (mTOR) pathway in rat intestinal epithelial [12]. Gln deprivation also induces autophagy and alters the mTOR and mitogen-activated protein kinase (MAPK) signaling pathways. Therefore, we hypothesized that a Gln-autophagy regulatory axis might be the potential key factor in UEC progression.

In the past decade, several studies have provided evidence that autophagy was associated with cancers. In tumor microenvironment, autophagy is a double-edged sword, acting as both a tumor suppressor that selectively degrades proteins and damages organelles and at the same time a cell-surviving promoter that accelerates tumor growth. Some signaling pathways regulate not only autophagy but also tumorigenesis. For human UEC, isoliquiritigenin (ISL), a licorice flavonoid, plays an anti-tumor role by inducing autophagy, indicating that autophagy can be used as a therapeutic agent [13]. Deng et al. have found that a novel estrogen-induced gene *EIG121* regulates autophagy and promotes cell survival [14]. Similarly, knockdown of estrogen receptor-α induces autophagy [15], suggesting that there should has a potential link between estrogen, metabolism and autophagy. However, the mechanisms of estrogen-mediated autophagy in UEC are poorly unknown [16].

Base on the correlations between estrogen, metabolism and UEC, the aim of this study is to investigate the relationship between estrogen and Gln metabolism and their roles in UEC progression in vitro and in vivo, and explore potential medicines for targeting estrogen/Gln metabolism in UEC cell (UECC).

## Methods

### UECC lines culture

UECC lines, Ishikawa and KLE cells, were obtained from the Shanghai Research Center for Model Organisms (Shanghai, China). Both of cell lines were cultured in DMEM/F12 (Gibco, Auckland, NZ) supplemented with 1% penicillin-streptomycin (HyClone, Utah, USA) and 10% certified FBS-charcoal-stripped (Biological Industries, Israel) and maintained at 37 °C with 5% CO_2_.

### Cell viability assays

The cell viability was examined by Cell Counting Kit-8 (CCK-8) assay (Dojindo, Tokyo, Japan) according to the manufacturer’s specifications. All the experiments were conducted and verified for at least three times.

### Western blotting

The samples were homogenized in 0.1% SDS buffer containing 10 mM EDTA, 125 mM NaCl, 25 mM HEPES, 0.5% deoxycholic acid, 10 mM Na_3_VO_4_, 0.1% SDS, 1% Triton X-100 with Complete™ protease inhibitor cocktail (Roche, Basel, Switzerland). The cell lysate was centrifuged at 12,000 rpm for 15 min. Then the supernatant-contained protein was collected and the protein concentration was tested by protein assay kit (Bio-Rad, CA, USA). The collected protein was separated on SDS-PAGE gel, and transferred onto PVDF membrane (Millipore, MA, USA). The membrane was blocked with 5% skim milk for 1 h to reduce non-specific binding. Then, the membrane was incubated with one of the following rabbit polyclonal primary antibodies: anti-LC3B-I&II, anti-Beclin-1, anti-p62, anti-β-actin, anti-Tublin, anti-C-myc, anti-N-myc, anti-L-myc, anti-GLS and anti-ERα (Cell Signaling Technology, MA, USA) at 4 °C for 12 h. After 3 times of washes, the blot was incubated with secondary antibody HRP-conjugated goat anti-rabbit IgG (Cell Signaling Technology, USA) for 1 h at room temperature. Finally, the signal was detected by the enhanced chemiluminescence kit (Biorbyt, CA, USA) and exposed to X-film

### Quantitative real-time polymerase chain reaction (qRT-PCR)

The cells were collected to extract total RNA by Trizol and then 500 ng of RNA was reverse-transcribed in accordance with the specification of FastKing RT Kit (TIANGEN, Beijing, China). According to the gene sequences, Primer 5.0 was used to design the primers, which were produced by Shanghai Sangon Biological Engineering Technology & Services Company (Shanghai, China) (Table 1). The reaction conditions of qRT-PCR were as follows: operating at 95 °C for 15 min once and then 40 cycles under 95 °C for 30 s, 60 °C for 45 s, 72 °C for 1 min. The reaction system was as follows (25 μl): 12.5 μl of Premix Ex Taq or SYBR Green Mix, 1 μl of forward primer, 1 μl of reverse primer, 1–4 μl of DNA template and ddH_2_O (TAKARA, Beijing, China). The relative quantification (RQ) of target genes was calculated by using the following formula: RQ = 2^-ΔΔCt^, and the result was used for statistical analysis.

**Table 1:** Primers in the study

Gene name	Forward primer	Reverse primer
Glutaminase	-TGACAAGATGGGCAACAGTG-	-GTTATTCCACCTGTCCTTGG-
c-MYC	-CGTCCTCGGATTCTCTGCTC-	-CTTCGCTTACCAGAGTCGCT-
n-MYC	-AGTTTGACTCGCTACAGCCC-	-CCCTAGCACTGCCTCCAAAA-
l-MYC	-CGAGTCGTAGTCCATGTCCG-	-GTTGGGGAGGAACGAGAGC-
GAPDH	-GT CGCCAGCCGAGCCACATC-	-CCAGGCGCCCAATACGACCA-

### Enzyme-linked immunosorbent assay (ELISA)

The detection of the Gln in the supernatant was carried out base on the specification of Gln ELISA kits (DAKEWEI, Beijing, China) and the steps were as follows: dilute the samples and add them to wells for tests; incubate at 37 °C for 30 min, then remove the liquid in each well, add scrubbing solution and aspirate it after 30s. Repeat the above steps for 5 times. Dry the plate, add enzyme standard reagent (50 μl), incubate the plate at 37 °C for 30 min; aspirate the liquid and add the scrubbing solution, aspirate after 30 s. Repeat the above steps for 5 times. Next, dry the plate. Add color developing agent to each well for incubation at 37 °C in the dark condition for 15 min. At last add 50 μl of stop buffer. Use the blank well as the zero set. Detect the optical density (OD) value (450 nm) of each well within 15 min. The microplate reader was purchased from Bio-Rad (Bio-Rad Laboratories, CA, USA).

### Transmission electron microscopy (TEM) of autophagy

Ishikawa and KLE were collected and then fixed in 2.5% glutaraldehyde, post-fixed in 1% osmium tetroxide. Samples were dehydrated in an ascending series of alcohols, and then embedded in epoxy resin. The ultrathin sections were cut, stained with uranyl acetate and lead citrate, and examined under a Philips CM120 transmission electron microscope (Philips, Amsterdam, Netherlands).

### Flow cytometry

A total of 1 × 10^6^ cells were resuspended in PBS buffer and incubated for 5 min at 4 °C. Then the cells were incubated for another 30 min on ice in 50 μl of staining buffer with 1 μg/ml of relevant fluorochrome-conjugated Ki67 or matched isotype control antibodies. 2 mL of the 1x Permeabilization working solution (BioGems, NJ, USA) were added to each sample, which were then centrifuged at 300–400 x g at room temperature for 5 min. The supernatant was discarded and the above steps were repeated twice. The labeled cells were washed twice with cold PBS. The samples were analyzed by using a Beckman Cyan flow cytometer (Becton Dickinson, NJ, USA) and Cellquest software (Becton Dickinson).

### Animal model and treatment

All animal experiments were approved by the Animal Ethics Committee of Shanghai General Hospital and were implemented in accordance with the Guide for the Care and Use of Laboratory Animals. Pathogen-free four-week-old female nude mice were obtained from Slaccas Animal Laboratory (Shanghai, China). The steps were as follows: Construct Xenograft model by subcutaneous injection of Ishikawa cells (2 × 10^6^ in phosphate-buffered saline containing 50% Matrigel, *n* = 6 for each group). Implant estrogen pellets (60-d time release, 0.72-mg β-estradiol/pellet; Innovative Research of America) subcutaneously unless otherwise noted. Formulate CB-839 (MedChemExpress, NJ, USA) solution with a concentration of 20 mg/mL in vehicle. The vehicle consists 25% hydroxypropyl-β-cyclodextrin (HPBCD; Roquette, Beinheim, France) in 10 mmol/L citrate; and pH is 2. The dose volume for all groups is 10 mL/kg. When the volume of tumors reaches approximately 100–150 mm^3^, dose the mice orally twice a day (every 12 h) with the vehicle or the 200 mg/kg CB-839 prepared in vehicle. Take records of the volume of tumors every 3 days after transplantation: tumor volume = length×width^2^ / 2. Record the tumor weight and profile after sacrificing.

### Confocal laser scanning microscopy

1 × 10^6^ treated Ishikawa cells were in a flat-bottom 24-well plate with cover glasses at the bottom. Cells were fixed with 4% v/v paraformaldehyde, permeabilized with 0.1% v/v Triton X-100 (Sigma, NJ, USA) and blocked them with 10% BSA in PBS. The cells were incubated with mouse anti-human GLS antibody (5 μg/ml, Abcam, MA, USA) at 4 °C overnight. Then the cells were incubated again with Alexa Fluor 488-conjugated anti-mouse secondary antibodies (1:1000; Cell Signaling Technology). The nuclei were stained with 4′,6-diamidino-2-phenylindole (DAPI; Beyotime, Shanghai, China). The images were captured by using a confocal microscope (Leica, Munich, Germany).

### Statistical analysis

All experiments were performed in triplicate and results represented at least three independent experiments. Student’s *t*-test was used to analyze two groups of data, and then one-way ANOVA was used to analyze the data of multiple groups. Data were expressed as means ± SD and results *P* < 0.05 were considered to be statistically significant.

## Results

### Estrogen promotes cell viability and inhibits the autophagy of estrogen-sensitive UECC

**Fig. 1:**
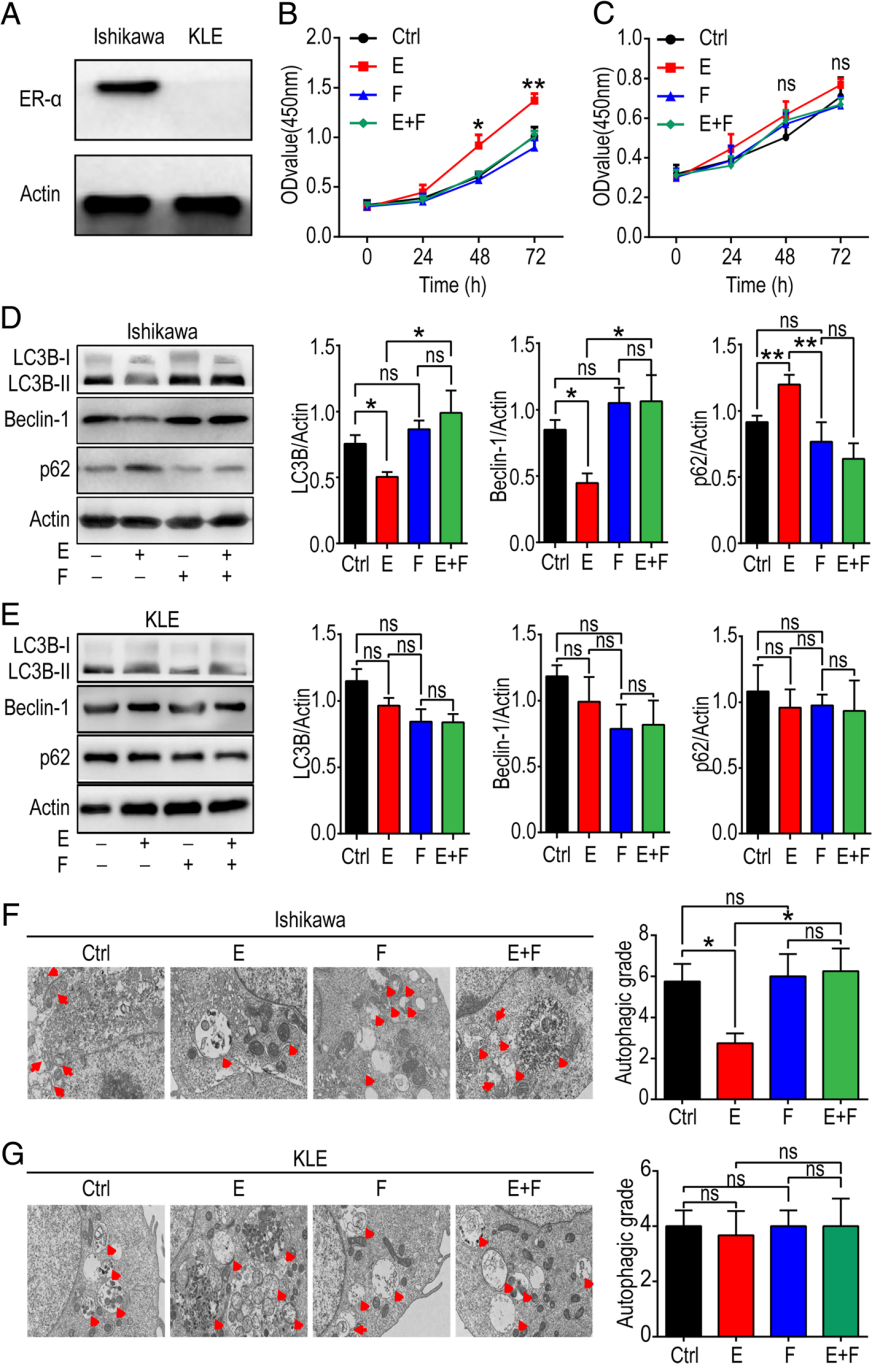
Estrogen enhances cell viability and inhibits autophagy of estrogen-sensitive UECC. **a** Western blotting image of ERα in Ishikawa and KLE cells. **b**, **c** Ishikawa and KLE cells were incubated with estrogen (10 nM) and/or fulvestrant (250 nM) for 24, 48 or 72 h and CCK-8 assay was performed to test cell viability. (Ishikawa group: estrogen-treated vs control, *P =* 0.010 at 48 h; *P =* 0.006 at 72 h). **d**, **e** Western blotting assay was carried out to analyze the level of autophagy-related proteins, LC3B, p62 and Beclin-1 after exposure with estrogen (10 nM) and/or fulvestrant (250 nM) for 48 h. Gray scale was analyzed by image J software (right). **f**, **g** After stimulation with estrogen (10 nM) and/or fulvestrant (250 nM) for 48 h, autophagic capacity of these cells was evaluated by TEM. The autophagic level was presented as APs and ALs per visual field. The data are presented as mean ± SD. *P < 0.05, **P < 0.01 and ns are interpreted as of no statistical significance. Ctrl: control group, E: estrogen-treated group, F: fulvestrant-treated group. E + F: estrogen plus fulvestrant-treated group

Firstly, we detected the protein level of ERα in Ishikawa and KLE cells, and confirmed ERα-positive Ishikawa as an estrogen-sensitive cell line while KLE as estrogen-insensitive because of absent expression of ERα (Fig. 1a). To investigate the biological effect of estrogen on Ishikawa and KLE cells, 10 nM estrogen and 250 nM fulvestrant (a selective estrogen receptor antagonist) were used to treat Ishikawa and KLE cells. As shown, exposure to estrogen promoted the viability of Ishikawa cells, and this process could be inhibited by fulvestrant; while estrogen had no significant effect on KLE cells (Fig. 1b, c). Then we tested the influence of estrogen on the autophagy capacity of Ishikawa and KLE cells. The levels of autophagy positively-related proteins, LC3B and Beclin-1 decreased while the level of p62, an autophagy negatively-related protein increased in Ishikawa cells after treatment with estrogen (Fig. 1d), but these effects could be reversed by fulvestrant (Fig. 1d). No obvious effect was observed in KLE (Fig. 1e). To further verify our results, TEM was used to examine autophagosomes (APs) and autolysosomes (ALs) in Ishikawa and KLE cells. APs and ALs accumulated markedly in Ishikawa but not in KLE cells after estrogen treatment (Fig. 1f, g), fulvestrant could also abrogate the inhibitory effect on autophagy of Ishikawa cells induced by estrogen (Fig. 1f, g). These data suggest that fulvestrant can profoundly relieve autophagy-suppression and proliferation-promotion induced by estrogen in estrogen-sensitive UECC in vitro.

### Estrogen promotes Gln metabolism of estrogen-sensitive UECC

**Fig. 2:**
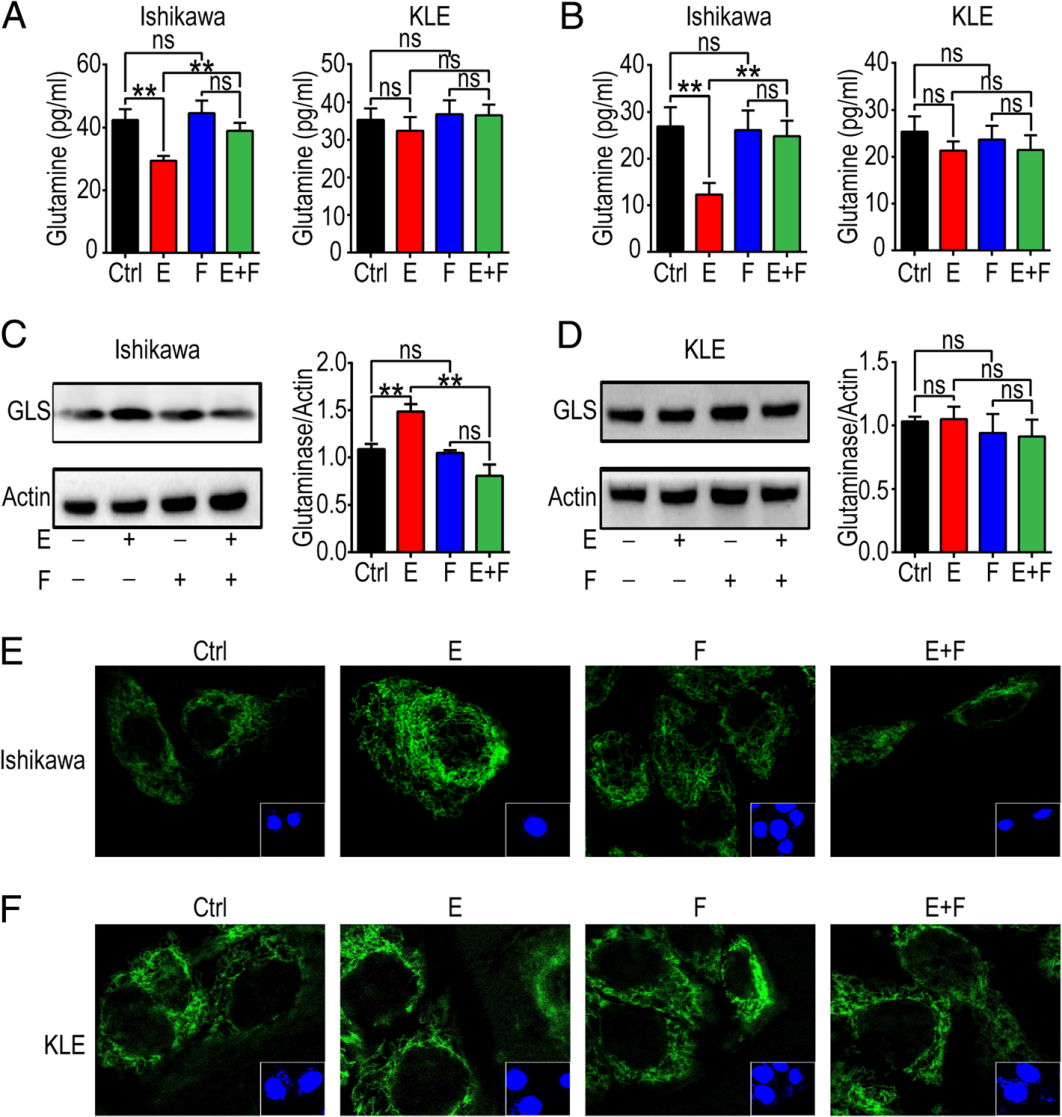
Estrogen stimulates Gln metabolism by up-regulating the expression of GLS. After treatment with estrogen (10 nM) and/or fulvestrant (250 nM) for 48 h, Ishikawa and KLE cells and supernatant were collected. **a**, **b** ELISA assay was used to test Gln in supernatant (**a**) and lysis solution (**b**). **c**, **d** Western blotting was carried out to evaluate the expression of GLS in protein. **e**, **f** Immunofluorescence of GLS (green) in Ishikawa and KLE was analyzed by laser confocal scanning microscope. The data are expressed as mean ± SD. **P < 0.01

To investigate the effect of estrogen on Gln metabolism, we treated Ishikawa and KLE with estrogen and/or fulvestrant for 48 h and found that estrogen treatment increased the uptake and consumption of Gln in the supernatant (Fig. 2a) and cell lysis (Fig. 2b) of Ishikawa but not KLE cells. Fulvestrant treatment effectively inhibited this process (Fig. 2a, b). Compared to the control group, the results of western blot showed that there was high level of GLS in estrogen-treated Ishikawa cells, and fulvestrant also abrogated it (Fig. 2c). However, the expression of GLS in KLE cells was not influenced by estrogen or/and fulvestrant (Fig. 2d). To further confirm this conclusion, confocal microscopy was used to detect the level of GLS in Ishikawa and KLE after estrogen and/or fulvestrant treatment, and the results were consistent with western blotting assay as expected (Fig. 2e, f). These findings indicate that estrogen promotes Gln metabolism in estrogen-sensitive UECC by up-regulating the level of GLS.

### Estrogen-induced activation of GLS in UECC is dependent on c-MYC

**Fig. 3:**
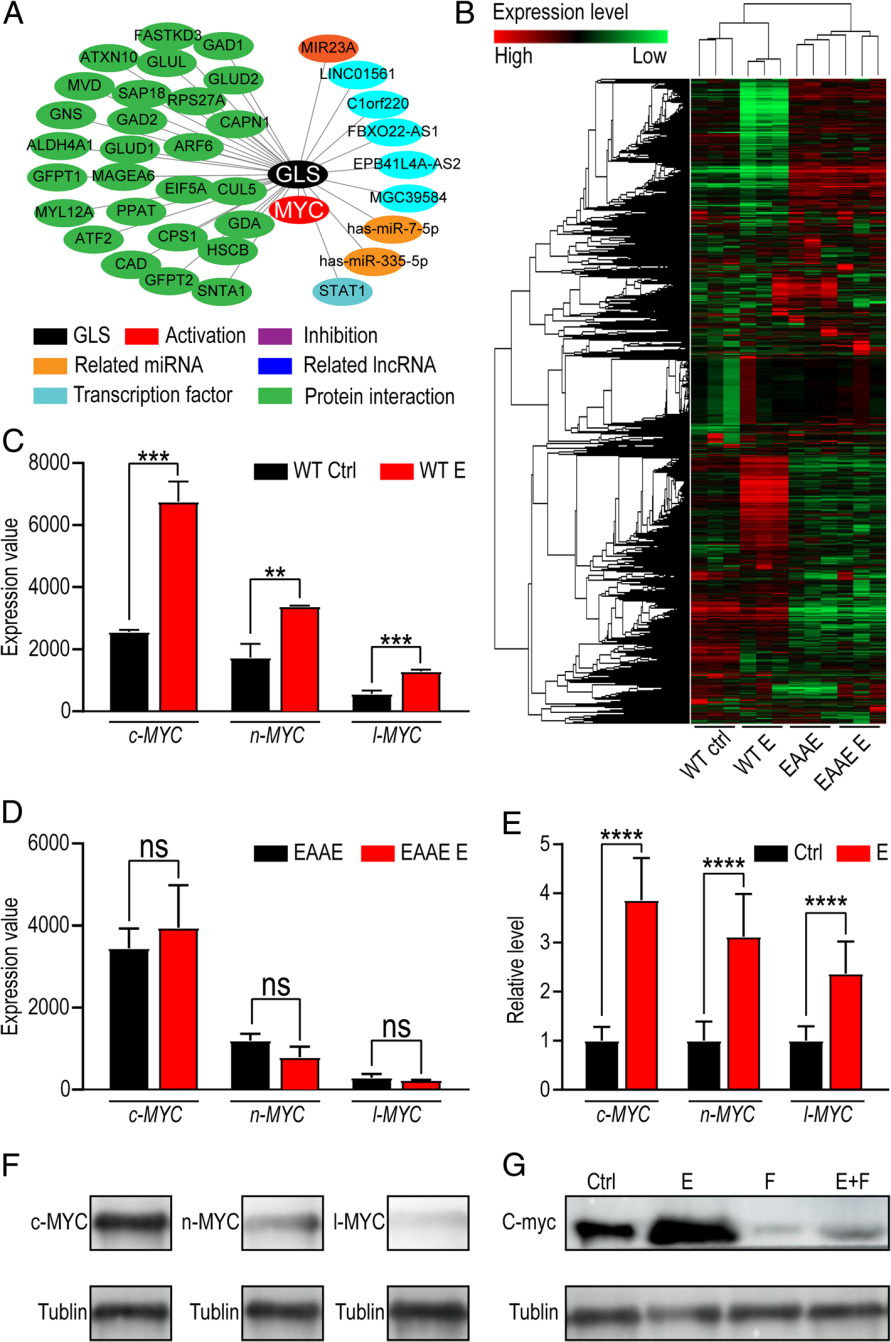
Estrogen-induced GLS in UECC is dependent on c-MYC. **a** Bioinformatics analysis was applied to predict regulatory factors associated with GLS. **b** Gene-array heat map of WT mice and EAAE mice models treated with or without estrogen. **c**, **d** MYC (c-MYC, n-MYC and l-MYC) levels of WT mice (**c**) and EAAE mice (**d**) with or without estrogen treatment. **e** c-MYC, n-MYC and l-MYC levels of Ishikawa cells with or without estrogen treatment were detected by qRT-PCR. **f** Protein levels of MYC (c-MYC, n-MYC and l-MYC) in Ishikawa cells were tested by western blot. **g** After stimulation with estrogen (10 nM) and/or fulvestrant (250 nM) for 48 h, western blotting assay was used to evaluate the expression of GLS in Ishikawa cells. The data are expressed as the mean ± SD. **P < 0.01; *** *P* < 0.005; ****P < 0.001 and ns are regarded as of no statistical significance

To explore the mechanism of estrogen on GLS in UECC, the bioinformatics analysis was used to analyze potential regulatory factors of GLS level. GCBI database (www.gcbi.com.cn) shows that MYC protein is the reliable activator of GLS (Fig. 3a). To verify whether MYC is the intermediate factor between estrogen signal and GLS, we analyzed gene microarray from GEO database (GSE56423), and differential expressed gene were reflected before and after estrogen treatment (Fig. 3b). As shown, three MYC subtypes (c-MYC, n-MYC and l-MYC) were increased after estrogen treatment (Fig. 3c). EAAE mouse has more severe mutations in the DNA-binding domain and lacks responses to estrogen. For EAAE mice, estrogen had no significant effect on c-MYC, n-MYC and l-MYC (Fig. 3d). To confirm these Bioinformatics analysis results, qRT-PCR was conducted and the results showed that c-MYC, n-MYC and l-MYC levels were increased in Ishikawa cells after treatment with estrogen (10 nM) for 48 h (Fig. 3e). In terms of protein level, there was abundant c-MYC and less level of n-MYC in Ishikawa cells, however, l-MYC was scarcely detected by western blotting (Fig. 3f). Of note, estrogen up-regulated c-MYC expression in Ishikawa cells, while this effect could be reversed by fulvestrant (250 nM) (Fig. 3g). These data indicate that estrogen increases the level of GLS by up-regulating c-MYC.

**Fig. 4:**
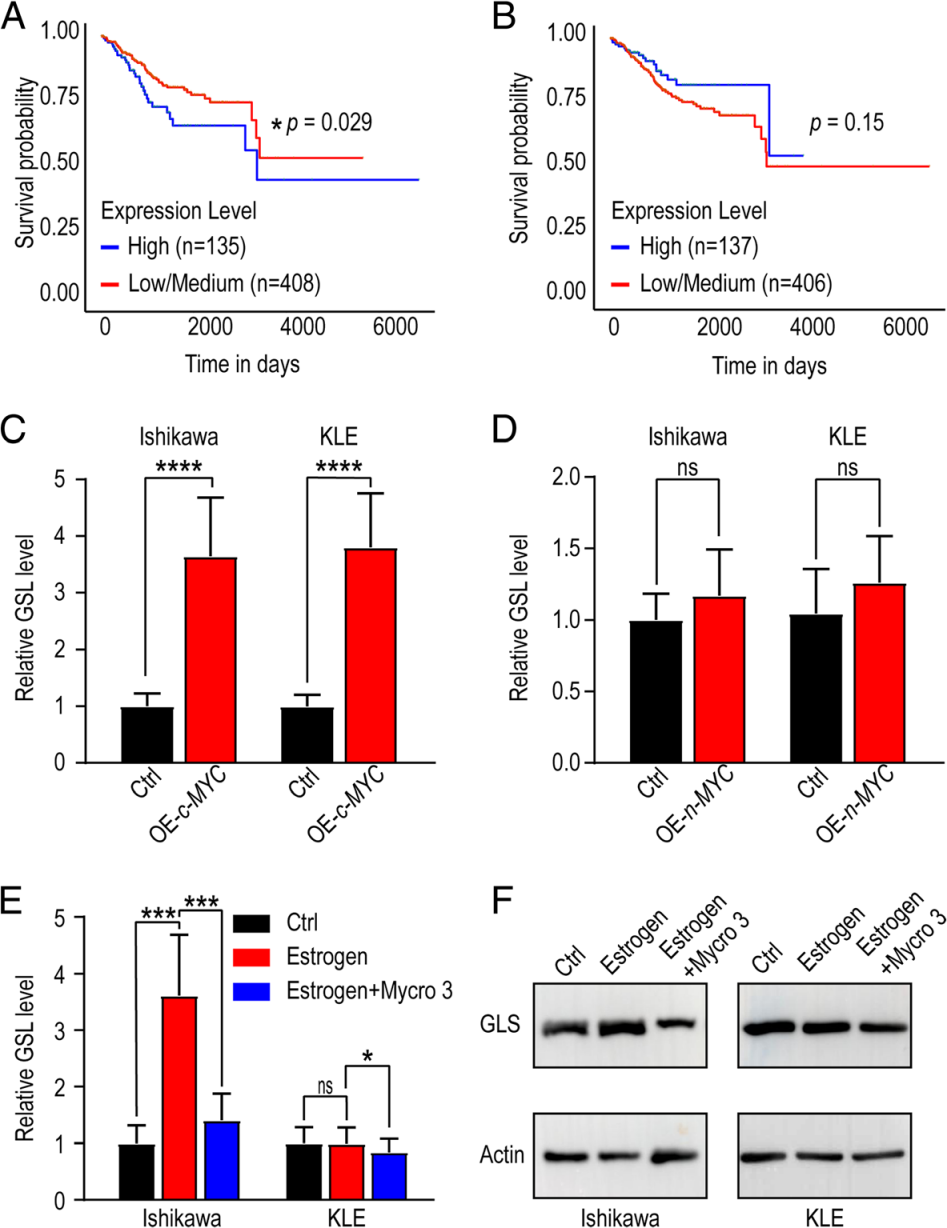
Estrogen-upregulated c-MYC is a crucial GLS promoter. **a**, **b** Based on TCGA database (https://tcga-data.nci.nih.gov), survival time of UEC patients were analyzed referring to c-MYC (**a**) or n-MYC (**b**) expression. **c**, **d** GLS levels of c-MYC (**c**) or n-MYC (**d**) overexpressed Ishikawa and KLE cells were detected by qRT-PCR. **e** Ishikawa and KLE cells were treated with estrogen (10 nM) and/or Mycro3 (50 μM) for 48 h and then GLS levels were tested by qRT-PCR. **f** Protein levels of GLS in Ishikawa and KLE cells were detected by western blotting after treatment with estrogen and/or Mycro3 for 24 h. The data are expressed as the mean ± SD. *P < 0.05; *** *P* < 0.005; ****P < 0.001 and ns means no statistical significance. *OE-c-MYC*: c-MYC overexpressed cells; *OE-n-MYC*: n-MYC overexpressed cells

According to TCGA database (https://tcga-data.nci.nih.gov), the UEC patients’ survival time could be classified depending on different levels of c-MYC or n-MYC. As shown, the prognosis of the UEC patients with high level c-MYC was better than that of the patients with low/medium level c-MYC (Fig. 4a). However, there was no similar effect of n-MYC (Fig. 4b). To explore the potential effects of different subtypes of MYC on GLS expression, overexpression of c-MYC (*OE-c-MYC*) or n-MYC (*OE-n-MYC*) in Ishikawa and KLE cells were constructed by plasmids transfection, and then qRT-PCR was used to analyze the transcription level of GLS. As shown, overexpressed c-MYC rather than n-MYC significantly increased the transcriptional level of GLS in both Ishikawa and KLE cells (Fig. 4c, d). Further analysis of qRT-PCR and western blotting results showed that mycro-3 (50 μM), a potent and selective c-MYC inhibitor partially reduced the stimulatory function of estrogen on GLS (Fig. 4e), suggesting that c-MYC was an important factor in the estrogen-GLS regulatory axis (Fig. 4f).

### Gln metabolism stimulates cell viability and inhibits autophagy in UECC

**Fig. 5:**
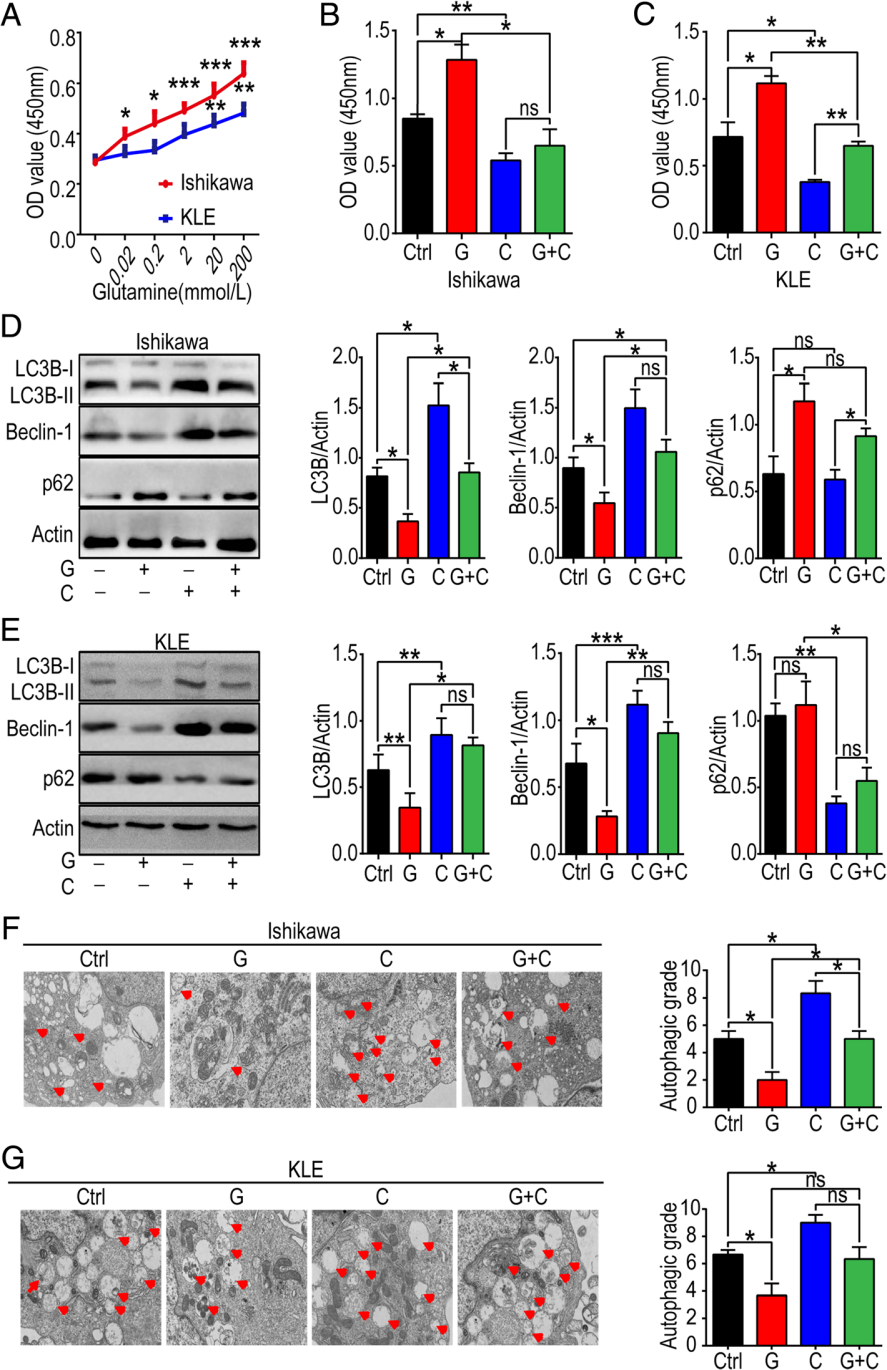
Gln metabolism promotes cell viability and inhibits autophagy of estrogen-sensitive and insensitive UECC. **a** Different concentrations (0, 0.02, 0.2, 2, 20, 200 mmol/L) of Gln were used to treat Ishikawa and KLE, and then CCK-8 assay was used to analyze cell viability. **b**, **c** Ishikawa and KLE cells were incubated with Gln (200 mmol/L) and/or CB-839 (1 μmol/L) for 48 h, and then cell viability was evaluated by CCK-8 assay; **d**, **e** the expression of autophagy-related proteins (LC3B, Beclin-1 and p62) was evaluated by western blotting, and gray scale of LC3B, Beclin-1 and p62 was analyzed by image J software; **f**, **g** In addition, TEM was performed to test autophagic degree of Ishikawa and KLE cells after treatment with Gln and/or CB-839. The autophagic degree was presented as APs and ALs per visual field. The data are expressed as the mean ± SD. *P < 0.05, **P < 0.01 or ***P < 0.001 and ns means of no statistical significance. Ctrl: control group, G: Gln-treated group, C: CB-839-treated group, G + C: Gln plus CB-839-treated group

To observe the effect of Gln on UECC, various concentrations (0-200 mmol/L) of Gln were used to treat Ishikawa and KLE cells. Cell viability gradually increased in Ishikawa and KLE cells with the elevation of Gln concentration (from 0 to 200 mmol/L) (Fig. 5a-c). In contrast, CB-839 (1 μmol/L), a potent selective oral bioavailable inhibitor of GLS decreased the cell viability of Ishikawa and KLE cells, and reversed the effect of Gln (Fig. 5a-c). Further investigation showed that exposure to Gln inhibited the expression of Beclin-1, LC3B, and up-regulated the expression of p62 in Ishikawa and KLE cells, these effects also could be reversed by CB-839 (Fig. 5d, e). In addition, the analysis of TEM echoed the results above (Fig. 5f, g). These data suggest that CB-839 can suppress cell viability and induce the autophagy of UECC by blocking Gln metabolism in vitro. Notably, this effect is not limited to estrogen-sensitive UECC.

### CB-839 induces autophagy and inhibits proliferation of estrogen-sensitive and insensitive UECC

**Fig. 6:**
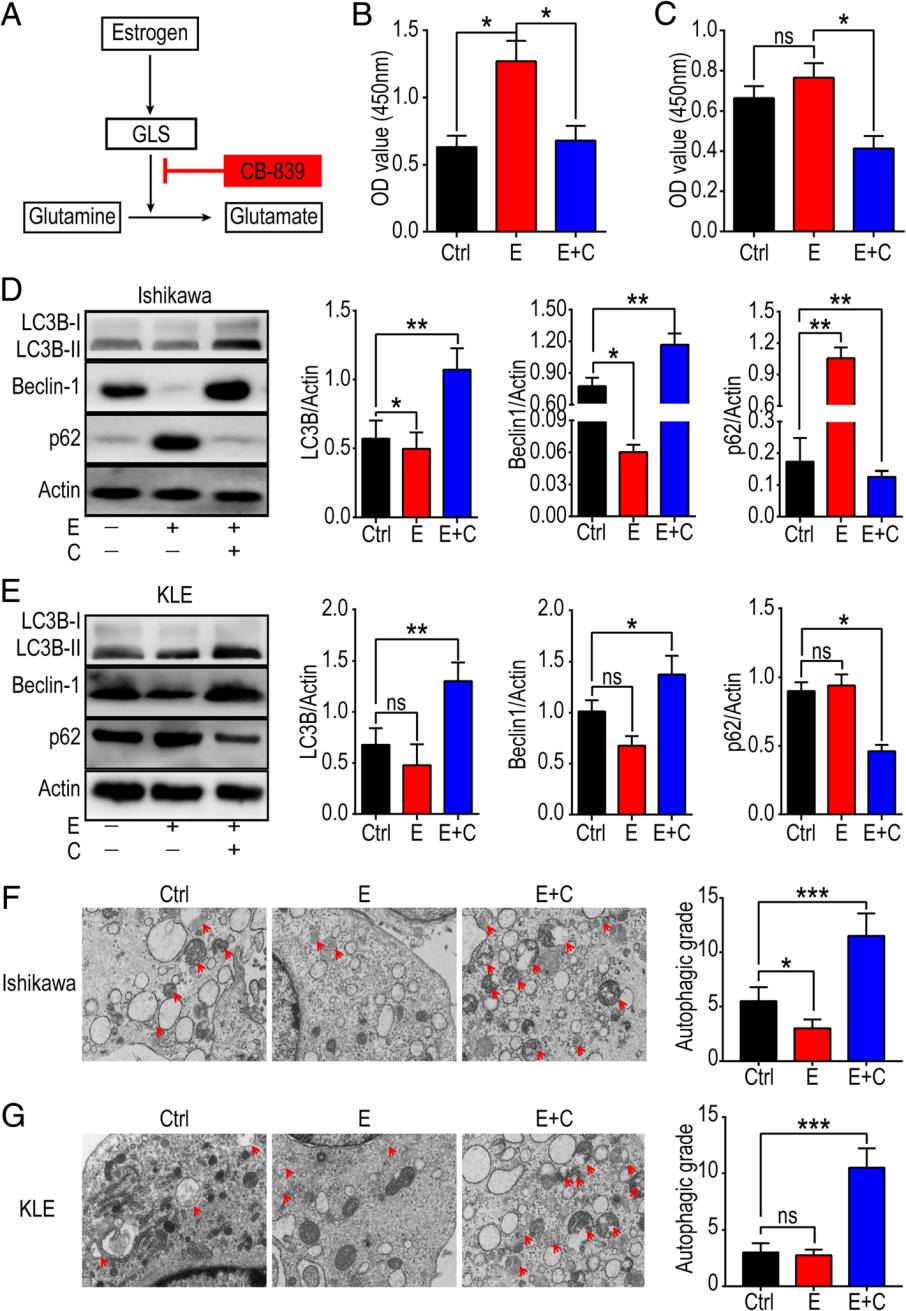
CB-839 induces autophagy and inhibits proliferation of estrogen-sensitive and insensitive UECC. **a** Gln metabolism model diagram. **b-g** After stimulation with or without estrogen (10 nM), or estrogen (10 nM) plus CB-839 (1 μmol/L) for 48 h, cell viability (**b**, **c**), the expression of autophagy-related protein, LC3B, Beclin-1 and p62 (**d**, **e**), and the autophagic level (**f**, **g**) of Ishikawa and KLE cells were analyzed by the CCK-8, western blotting, and TEM analysis. Gray scale of protein expression was analyzed by image J software (**f**, **g** right). The data are expressed as the mean ± SD. *P < 0.05, or **P < 0.01 and ns means no statistical significance. E: estrogen-treated group, C: CB-839-treated group, E + C: estrogen plus CB-839-treated group

To further investigate whether CB-839 regulate the biologic function of estrogen by inhibiting Gln metabolism (Fig. 6a), Ishikawa and KLE cells were incubated with or without estrogen, or estrogen plus CB-839, and the results showed that the combined exposure to estrogen and CB-839 completely reversed the regulatory effects of estrogen on promoting cell viability, enhancing expression of Beclin-1, LC3B and p62, as well as suppressing autophagy in Ishikawa cells (Fig. 6b, d, f). It’s remarkable that CB-839 also significantly suppressed cell viability and enhanced autophagy of KLE cells (Fig. 6c, e, g), suggesting that CB-839 plays a regulatory role in cell viability and autophagy of both estrogen-sensitive and insensitive UECC in vitro.

### CB-839 inhibits estrogen-stimulated UEC growth in vivo

**Fig. 7:**
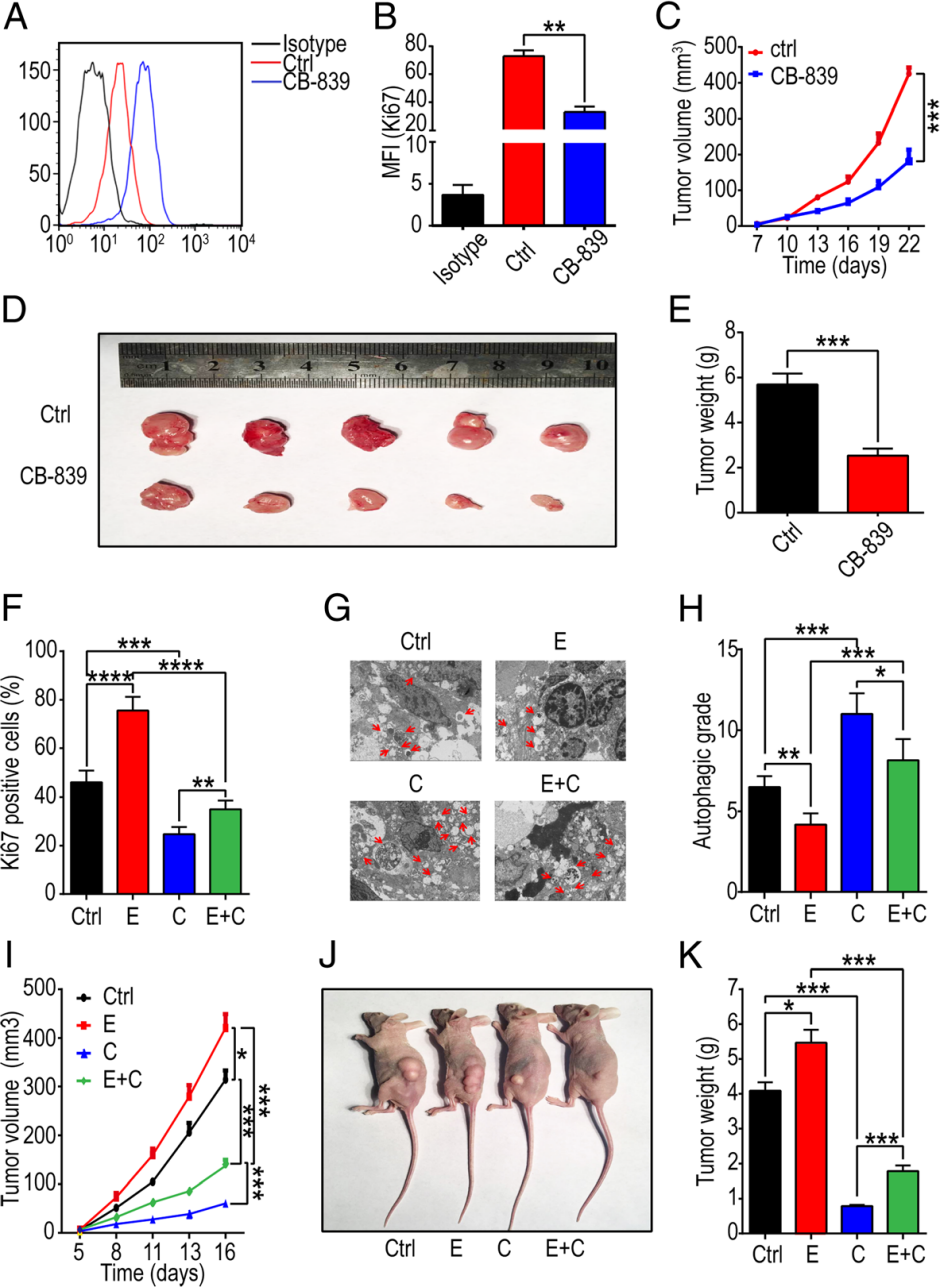
CB-839 treatment undermines estrogen-induced UEC growth in vivo. UEC xenograft was constructed in nude mice by subcutaneously injecting Ishikawa cells. **a**, **b** The mean fluorescence intensity (MFI) of Ki67 (a proliferation-related molecule) in UEC of control and CB-839-treated groups, was evaluated by flow cytometry. **c** The tumor volume was measured every 3 days. **d**, **e** Mice were sacrificed after 22 days and the weights of xenografts were recorded. **f** tumor tissues were separated digested, and then flow cytometry was used to reflect the MFI of Ki67 in cell suspension of UEC. **g**, **h** TEM was performed to analyze the autophagic degree, which was presented as APs and ALs per visual field. **i** Tumor volume was measured every 3 days. **j** Xenograft in mice. **k** Mice were sacrificed after 16 days and the weight of xenografts were measured. The data are expressed as the mean ± SD. *P < 0.05 or ***P < 0.001 and ns are regarded as of no statistical significance. E: estrogen-treated group, C: CB-839-treated group, E + C: estrogen plus CB-839-treated group

Considering the anti-tumor activation of CB-839 in vitro, we constructed UEC model in nude mice through subcutaneous injection of Ishikawa cells to further confirm it in vivo. As expected, the expression of Ki67 (a common cell proliferation-related molecule) in CB-839-treated group was weaker than that of control groups (Fig. 7a, b). In addition, CB-839 led to obvious reduction of tumor volumes and weights (Fig. 7c-e). Estrogen significantly elevated Ki67 level, decreased autophagy, accelerated the growth and increased the weight of UEC in mice (Fig. 7f-k), while CB-839 could obviously reverse these effects (Fig. 7f-k). This phenomenon points out a powerful anti-tumor effect of CB-839, which also inhibits estrogen-induced UEC growth in vivo.

## Discussion

Nowadays UEC is one of the most common invasive gynecological cancers worldwide. A lot of studies suggest that estrogen plays a crucial role in the development of UEC [17]. A variety of factors like aging, obesity, hepatic disease, and hyperthyroidism were found relating to increased generation of estrogen [18]. Moreover, several conditions, such as polycystic ovarian disease (PCOS), functional and non-functional tumors of ovary are associated with increased androstenedione production, which can stimulate peripheral estrogen formation. Furthermore, the relations between null parity and increased risk of endometrial and breast cancer may reflect the fact that many nulliparous women are more vulnerable to UEC [19]. Increased or prolonged exposure to estrogen of the uterus is associated with higher risk of UEC development.

Despite that UEC was already categorized as a genetic disease, actually it also can be considered as a metabolic disease. Altered metabolism in cancers leads to enhanced nutrient acquisition and facilitates metabolism of substances and energy such as amino acid. The net effects of these activities are to support cell growth, proliferation and autophagy and so on [20]. In our study, we hypothesized that metabolism regulation is involved in the process of estrogen-induced progression of UEC. Evidence has been provided that estradiol could stimulate proliferation of endometrial cancer cells of estrogen-sensitive UEC cell line Ishikawa in the absence of serum or added growth factors [21]. Here, we reported that estrogen activated Gln metabolism in estrogen-sensitive UECC, and this effect was dependent on up-regulation of GLS, suggesting that the link between estrogen and GLS indeed exists.

GLS, a key metabolic enzyme, is regulated by multiple mechanisms. Transcription factor c-Jun was a key regulator of mitochondrial GLS levels. Activation of c-Jun downstream of oncogenic Rho GTPase signaling leads to elevated GLS gene expression and GLS activity [22]. Moreover, estrogen-related receptor α (ERRα) is co-activated by peroxisome proliferator-activated receptor gamma coactivator (PGC)-1α, a crucial regulator for Gln metabolism genes in ERBB2^+^ breast cancer [23]. In this study, treatment with CB-839 could inhibit cell viability of both estrogen-sensitive and insensitive UECC in vitro, and significantly abolished estrogen-induced growth of UECC. It has been reported that estrogen could induce c-MYC proto-oncogene expression in UECC. c-Myc was reported to selectively bind to the promoter regions of high-affinity Gln transporters ASCT2 and SN2, thus promoting glutaminolysis in mouse embryonic fibroblasts [24]. Of note, we found c-MYC not n-MYC or l-MYC activated the transcription of GLS under the regulation of estrogen.

The primary activation of GLS is to catalyze the hydrolysis of Gln to glutamate. As glutamate can further convert to α-ketoglutarate by the glutamate dehydrogenase (GDH), then α-ketoglutarate can be utilized directly in the citric acid cycle for generating energy. Another important function of Gln metabolism is to provide precursors for glutathione production, which helps to maintain the oxidative status of cells. Indeed, GLS is directly linked to redox balance in cancer cells [25]. Therefore, Gln and its metabolites can provide raw materials for cell growth and enhance cell proliferation and growth. Here, we found that exogenous Gln supplement accelerated the growth of both estrogen-sensitive cell line Ishikawa and insensitive cell line KLE. Moreover, we observed that Gln inhibited autophagy in Ishikawa cells.

Since Gln inhibited autophagy and enhanced the growth of both Ishikawa and KLE cells, we hypothesize that estrogen is not a specific upstream regulatory factor of GLS activity. Increased levels of TGF-β1 are observed in many human tumors, including UEC, and are associated with poor clinical outcomes [26, 27]. Specially, TGF-β1 stimulates migration of type II endometrial cancer cells by down-regulating PTEN via activation of SMAD and ERK1/2 signaling pathways [28]. TGF-β1 can up-regulate the expression of GLS1 in myofibroblasts through both SMAD3- and p38 MAPK-dependent signaling pathways [29]. Therefore, TGF-β1 might be another modulatory factor of active Gln metabolism in endometrial cancer, especially in the estrogen-independent type. This effect and mechanisms need our further study.

In terms of the links between amino acid and cell autophagy, Mortimore and Schworer in 1977 reported that amino acid deprivation induced the accumulation of autophagosomes in perfused rat liver [30]. In addition, a simple combination of alanine and leucine (Leu) mimics the inhibitory effect on autophagy in near-physiological concentrations [31]. Combinations of low concentrations of leucine and proline, Gln, or asparagine also effectively play as inhibitors of cell autophagy [32]. Actually, amino acid metabolism is one of the critical factors in the autophagy process in multiple pathways. Fogel et al. reported that Beclin 1-S91 and S94 might also be sensitive to amino acid starvation pathways [33]. Recent studies have provided mechanistic insight into the stimulatory effects of Leu, Gln and arginine (Arg), which are three of the most potent amino acids that activate mTORC1 via distinct sensing pathways [34]. Gln deprivation is also reported to cause cell autophagy [35]. As reported, Gln is transported through the concerted function of at least four different systems, some of which also provide service for the transport of other amino acids. For these mechanisms, there is redundant, ubiquitous and thus robust maintenance of cytoplasmic Gln across the broad cell environment [36]. In HeLa, HCT-116, A549, PC3 and DU145 cells, Gln deprivation can increase autophagic activity by elevating levels of the autophagosome-associated form of LC3 (LC3-II) and decreasing autophagy-degraded protein p62 [37]. Our recent studies have confirmed that estrogen can significantly restrict autophagy of human endometrial stromal cells [38, 39]. In this study, we found that estrogen activated Gln metabolism, which further inhibited autophagy in Ishikawa but not in KLE cells. And CB-839 treatment enhanced cell autophagy by inhibiting enzymatic activity of GLS.

Indeed, it has been years since people started to consider to use the inhibition of glutaminolysis for cancer therapy [40]; however, there is still no encouraging news. One limitation lies in the fact that GLS is not only required for metabolism in cancer cells, but also for the development, growth, and physiological functions maintenance of normal tissues. Thus, inhibiting glutaminolysis may result in serious complications in patients. Indeed, early preclinical studies with DON (6-diazo-5-oxy-L-norleucine) and with other Gln mimetic compounds (azaserine, acivicin) showed limited antitumor effects and severe toxicity (nephrotoxicity, gastrointestinal toxicity and myelosuppression) [41]. In addition, other compounds such as BPTES (an allosteric inhibitor of GLS) and 968 (an inhibitor of Rho GTPase) have been described. Although these compounds exhibit both increased specificity against GLS isoforms and antitumor effects on several cancer cell lines, their hydrophobic nature still hinders their application in vivo [42]. Notably, a recent promising inhibitor of GLS, CB-839, has currently being tested in clinical trials against several types of tumors [43]. Our study found that CB-839 effectively inhibited Gln metabolism, and abrogated estrogen-induced cell growth and autophagy suppression in estrogen-sensitive UECC in vitro and in vivo.

Autophagy is related to metabolism, death of cells, stress response and carcinogenesis. In the process of tumor development and during research for cancer therapy, autophagy has been reported to paradoxical functions since both cell survival and cell death. Autophagy can encourage cell adaptation and survival, but under some conditions it also leads to cell death [44]. There are two potential strategies: (1) induce autophagy and enhance its anti-tumor effect, and (2) inhibit autophagy and induce apoptosis. The first proposed strategy is based on the observation that autophagy is one of the antitumor effects of anti-cancer therapies [45]. Cisplatin-based chemotherapy frequently resulted in acquired resistance of cancer cells. The levels of LC3-related autophagy were significantly lower in cisplatin-resistant cells, and autophagosome formation was dramatically reduced in the resistant cells [46]. Therefore, cisplatin treatment combined with autophagy-induced treatment may achieve better effects. Specifically, inhibitors of the PI3K/Akt/mTOR pathway or overexpression of autophagy-inducing gene products such as PTEN and Beclin-1 are potentially feasible to treat tumor through autophagy pathway. Here we found that CB-839 treatment could inhibit the progression of UEC by inducing autophagy. Further investigations are required to clarify the biological role of autophagy and then its feasibility in the diagnosis and treatment of UEC.

## Conclusions

**Fig. 8:**
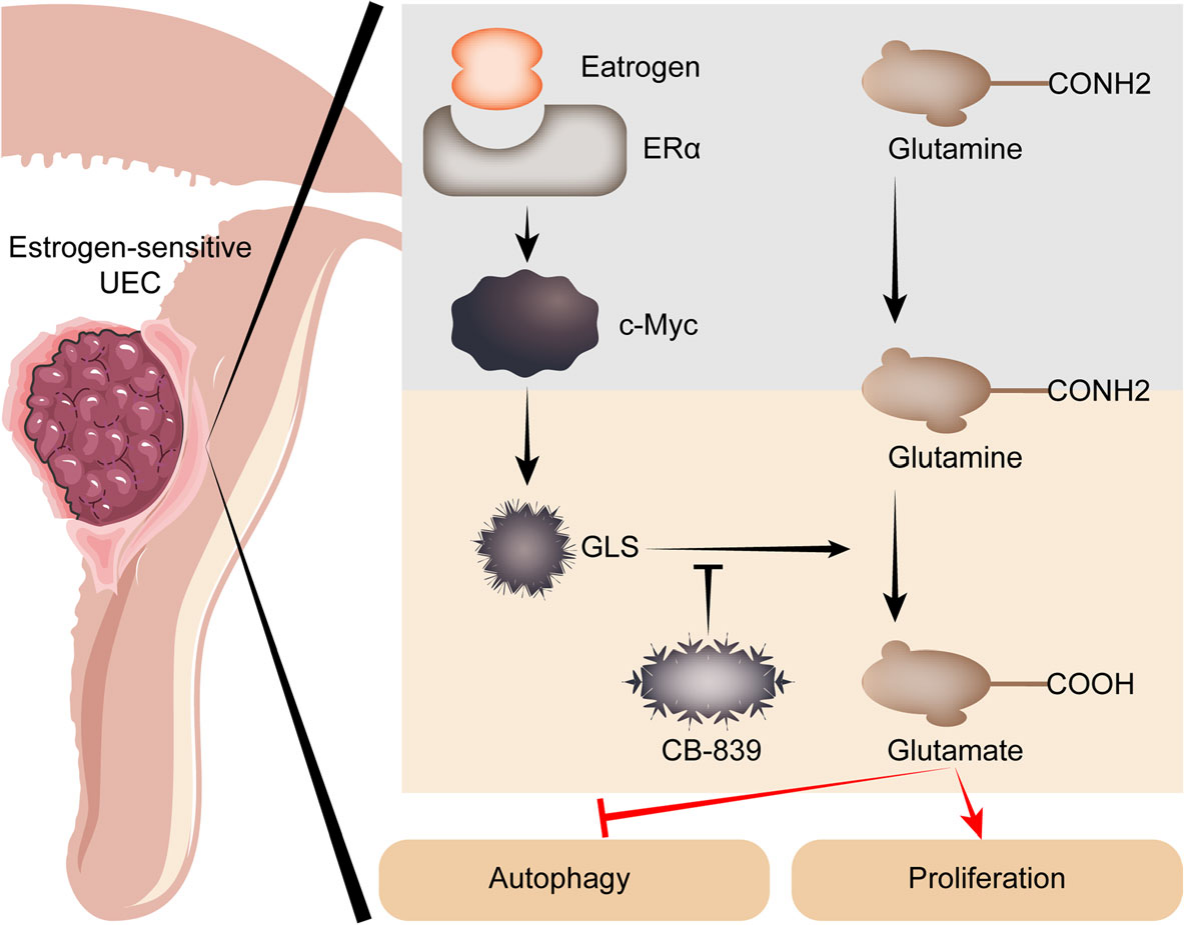
Schematic diagram of estrogen-Gln metabolism in estrogen-sensitive UECC. For estrogen-sensitive UEC, estrogen remarkably increases GLS and activates glutamine metabolism through up-regulating c-MYC. These processes remarkably lead to enhanced cell viability and decreased autophagy of estrogen-sensitive UECC. The GLS inhibitor CB-839 restricts the effects of estrogen on UECC’s growth and autophagy by inhibiting Gln metabolism

Collectively, as shown in Fig. 8, estrogen activates Gln metabolism in estrogen-sensitive UECC by up-regulating the levels of glutaminase (GLS), and this process is dependent on c-MYC. With the Gln metabolism-regulatory pathway, estrogen promotes cell viability and inhibits autophagy of UECC. The GLS inhibitor CB-839 inhibits cell viability and induces autophagy of estrogen-sensitive and insensitive UECC, also restricts the effects of estrogen on UECC’s growth and autophagy by inhibiting Gln metabolism in vitro *and* in vivo. These findings could provide scientific basis for future exploration of therapeutic strategies for UEC patients, especially with the abnormal Gln metabolism or autophagy.

## Data Availability

All data in our study are available upon request.

## Abbreviations

ALs: Autolysosomes
Aps: Autophagosomes
CCK-8: Cell Counting Kit-8
ELISA: Enzyme-linked immunosorbent assay
ER: Estrogen receptor
GDH: Glutamate dehydrogenase
Gln: Glutamine
GLS: Glutaminase
ISL: Isoliquiritigenin
TEM: Transmission electron microscopy
UEC: Uterine endometrial carcinoma
